# Immunity by Injection of Chemical Bodies

**Published:** 1887-08

**Authors:** 


### *IMMUNITY BY INJECTION OF CHEMICAL BODIES.*

Dr. L. C. Wooldridge recently communicated to the Royal Society a method by which he had been able to protect rabbits from anthrax, which is of considerable interest in connection with the general question of the nature of protection in this and other diseases depending on micro-organisms. The method consists in cultivating the anthrax bacillus in an alkaline solution of a peculiar proteid body which can be obtained from the testis and thymus gland. The growth is not abundant, and, after two days at 37° C., it is removed from the culture-fluid by filtration. A small quantity of filtered liquid is injected into the circulation of a rabbit, and the animal can then withstand the inoculation of extremely virulent anthrax blood. The bacillus itself grown in this peculiar culture-fluid has no protective influence; it either kills or it has no effect. The result is extremely curious, for hitherto protection against zymotic disease has been effected by the communication to the animal of a modified form of the disease against which protection is sought. In Dr. Wooldridge's experiments the protection must be produced by some chemical body, the product of the activity of the bacillus. The observation belongs to a new order of facts, and appears to fall in with M. Pasteur's theory as to the method in which immunity to hydrophobia is produced by inoculation of the spinal cord of rabid rabbits. Both find some support in Professor Cash's experiments with perchloride of mercury, in which it was shown that after animals had taken a sufficient quantity of the drug, they were no longer liable to anthrax.—*The British Medical Journal*, May 21, 1887.

